# Patients, parents and professional perspectives on molecular radiotherapy for neuroblastoma and paediatric neuroendocrine cancers

**DOI:** 10.1097/MNM.0000000000001956

**Published:** 2025-01-23

**Authors:** Lisa Whittaker, Leona Knox, Zara Aitchison, Connie Peet, Aine O’Donovan, Juliet Gray, Simon Wan, Glenn D. Flux, Mark N. Gaze

**Affiliations:** a Centre for Medical Engineering, King’s College London,; b Solving Kids’ Cancer,; c Neuroblastoma UK,; d Department of Radiotherapy, University College London Hospitals NHS Foundation Trust,; e Department of Paediatric Oncology, University Hospital Southampton, NHS Trust, Southampton,; f Department of Nuclear Medicine, University College, London Hospitals NHS, Foundation Trust; g Joint Department of Physics, The Royal Marsden NHS Foundation Trust & Institute of Cancer Research, and; h Department of Oncology, University College London Hospitals NHS Foundation Trust and UCL Cancer Institute, University College London, London, UK

**Keywords:** clinical trials, dosimetry, molecular radiotherapy, neuroblastoma, neuroendocrine cancer, Patient and Public Involvement and Engagement

## Abstract

Treatment with radioactive drugs (molecular radiotherapy, MRT) is an option for selected children with neuroblastoma and neuroendocrine cancers. As few hospitals are appropriately equipped and staffed to provide paediatric MRT, many families have to travel long distances from home for prolonged periods. To improve professional understanding of the challenges faced by children receiving these treatments and their parents, and to help them appreciate the difficulties faced by professionals in delivering complex treatments, a meeting bringing together parents, patients and professionals was held. Ten people (five parents of children with neuroblastoma, two parents of children with neuroendocrine cancers, two adults who had received treatment for neuroendocrine cancers in childhood and one adult treated for neuroblastoma) gave personal perspectives of treatment with MRT. Three professionals from different disciplines involved with this treatment and research to improve its results gave their views on the administration of MRT, and how treatment outcomes might be improved. Fifteen people, including parents and professionals, contributed to the general discussion. Following the meeting, a questionnaire was circulated to those attending to capture their overall views, and any reflections they may have had after the meeting. Whilst many positive comments and compliments were received, this report focuses on the reported challenges and difficulties. The event is an example of meaningful Patient and Public Involvement and Engagement and has resulted in development of better information resources, strategies to mitigate inconveniences experienced and a standing group of advocates to advise on research design and acceptability.

Molecular radiotherapy (MRT) is treatment with radioactive drugs, sometimes called radiopharmaceutical or radioligand therapy. MRT is a treatment option for carefully selected children with neuroblastoma, neuroendocrine cancers and thyroid cancer [1,2]. Although different vectors targeting physiological pathways are used, the actual oncological treatment is given with radiation, not the carrier. Its effectiveness varies. Thyroid cancer, even when metastatic, is sometimes cured [3,4]. In neuroblastoma, responses occur and survival may be prolonged, but cure is unusual [5]. Treatment is generally well tolerated, with limited side effects. As the drugs are radioactive, imaging after administration allows both qualitative evaluation of localisation and quantitative assessment of radiation dose delivery. Radiation dosimetry, the measurement of the absorbed radiation dose in tumour deposits and other tissues and organs, requires sequential scans after the radiopharmaceutical administration [6].

MRT may be more challenging for some families than other cancer treatments for several reasons. Parents are often concerned because of these, but many are reassured by clear information and talking with others.

Firstly, as the child becomes radioactive from a day or two to several weeks, depending on the treatment regimen used, patients must be isolated in a protected hospital suite to reduce radiation exposure to others. Contact from doctors, physicists and nurses is kept to the minimum necessary. Caregivers must limit the time spent in close contact with the patient, increase the distance between them and use barriers to keep their personal radiation exposure as low as reasonably achievable. They must use personal protective equipment including plastic aprons, overshoes and gloves to reduce the risk of contamination by radioactive liquids including urine, which may be more difficult in younger children who are not fully continent. Parents must give informed consent to be legally designated as comforters and carers, as radiation exposure is governed by legislation, and must be measured [7]. Radioactive children are not allowed contact with pregnant women or other children. This may delay discharge from hospital, depending on family circumstances, and also delay return to school.

Secondly, only three hospitals in the UK currently have the necessary facilities and expertise to give MRT safely to young children [8]. Similar limitations may be present in other countries. This means that patients and parents may need to be hundreds of miles from home, away from work and other children, for several weeks at a time, adding a financial burden to other anxieties [9].

Thirdly, accurate tumour dosimetry may be perceived as a burden, as it may require repeated general anaesthesia in younger children. However, this is less than with external beam radiotherapy where daily general anaesthesia may be required for 6 weeks or more.

Supply of radiopharmaceuticals is uncertain, and last-minute cancellations because of this may pose significant problems for patients, and rescheduling challenges for staff.

Finally, treatment schedules are not perfect: there is room for improvement. Offering patients entry into clinical trials where feasible is considered best practice. Selected trials in paediatric MRT are shown in Table 1.

**Table 1:** Selected recent, current and planned clinical trials of paediatric molecular radiotherapy in the UK and other countries

**Recent – published**
**LuDO:** A phase IIa trial of molecular radiotherapy with 177-lutetium DOTATATE in children with primary refractory or relapsed high-risk neuroblastoma. (Recruitment 2013–2017) [10] **NANT 2011-01:** Randomised phase II pick the winner study of ^131^I-mIBG, ^131^I-mIBG with vincristine and irinotecan, or ^131^I-mIBG with vorinostat for resistant/relapsed neuroblastoma. (Recruitment 2014–2019) [11]
**Recruitment completed – pending completion and publication**
**MINIVAN:** NCT02914405: Phase I study of 131-I mIBG followed by nivolumab & dinutuximab beta antibodies in children with relapsed/refractory neuroblastoma. (Recruitment 2018–2024)
**VERITAS:** NCT03165292: Evaluation of 2 intensification treatment strategies for neuroblastoma patients with a poor response to induction. (Recruitment 2018–2022 – terminated prematurely)
**mIBG With Dinutuximab ± Vorinostat:** NCT03332667: A phase I study of ^131^I-mIBG with dinutuximab ± vorinostat for relapsed/refractory neuroblastoma. (Recruitment 2018–2024)
**NETTER-P:** NCT04711135: Study to evaluate safety and dosimetry of lutathera in adolescent patients with GEP-NETs and PPGLs. (Recruitment 2022–2023)
**Current**
**LuDO-N:** NCT04903899: A phase II trial of a personalised, dose-intense administration schedule of 177-lutetium DOTATATE in children with primary refractory or relapsed high-risk neuroblastoma. (Open and recruiting)
**Neuroblu 02:** NCT03966651: A clinical study evaluating the safety of peptide receptor radionuclide therapy (PRRT) With 177Lu-DOTA0-Tyr3-Octreotate in children with refractory or recurrent neuroblastoma expressing somatostatin receptors. Conditions. (Open and recruiting)
**Planned**
**MINT:** A biomarker enriched phase I/II clinical trial of ^131^I-mIBG therapy with talazoparib for the treatment of relapsed and/or refractory neuroblastoma. (Funded, pending opening)

Given the specialist nature of these treatments, and the demands it places on patients and their parents, it is vital that professionals understand their experiences. Similarly, it may be beneficial for patients and parents to understand the professional procedures required to deliver treatment with accuracy and precision. To be meaningful, all stakeholders need to actively engage in this exchange of expertise [12].

Patient and Public Involvement and Engagement (PPIE) is increasingly being recognised as essential to optimise clinical services and support worthwhile research [13]. To understand the needs of children undergoing specialist cancer treatment, it seems obvious that one should ask those who know their needs best [14]. In paediatric oncology research, it is often the parents of children affected by cancer, who become actively involved in research. They are passionate advocates for their children and are often instrumental in voicing the needs of their child and in raising funds for research where funding may be limited [15].

In order to share perspectives and learn from one another a meeting was convened to bring together patients, parents and professionals with experience of paediatric MRT. The purpose was to have a two-way conversation, ensuring that patients and parents voices were listened to [16]. The aims were to identify ways to improve the quality of information and clinical service delivery, to demonstrate the need for more research and to create a standing group of experienced individuals to contribute to clinical trial development.

A one-day meeting was held in London, in June 2024, with a range of invited participants including healthcare professionals, parents, patients and charity representatives (Table 2). For reasons of practicality, only those treated at one centre were invited. Costs of attendance (travel and accommodation) were reimbursed as many had travelled long distances. The workshop commenced with brief introductory presentations from professionals to set the scene and to illustrate some of the professionally perceived challenges in delivering MRT treatment and research to children and young people. There followed talks from patients and parents with lived experience of MRT, reflecting on what went well, what could have been done better and suggestions to enable a better patient and family experience. These presentations were sympathetically facilitated by two bereaved parents of children with neuroblastoma who had not received MRT. Refreshment breaks provided a useful opportunity for dialogue, networking and informal discussion. The workshop concluded with a general discussion to identify common threads, and suggestions for improvement.

**Table 2:** Attendees at the workshop

**Speakers**
***Patients*** ** **Adults who were previously treated as children for neuroendocrine cancers (2) ** **Adult who was treated for neuroblastoma in adult life (1) ** **Parents of surviving children treated for neuroblastoma (3) ** **Bereaved parents of children treated for neuroblastoma (2) ** **Parents of surviving children treated for neuroendocrine cancers (2) ***Professionals*** ** **Clinical oncologist (1) ** **Nuclear medicine physicist (1) ** **Specialist therapeutic radiographer (1)
**Facilitators** ** **Bereaved parents of children with neuroblastoma (2) ** **Patient and Public Involvement and Engagement specialist (1)
**Others** ** **Accompanying parents (4) ** **Paediatric oncologist (2) ** **Paediatric oncology research nurse (1) ** **Nuclear medicine physician (1) ** **Nuclear medicine physicist (1) ** **Molecular radiotherapy researcher (1) ** **Hospital play specialist (1) ** **Charity representatives (3)

After the meeting, a brief questionnaire was distributed electronically to all participants, asking for their thoughts and impressions after reflecting on their experiences that day, and for ideas for further steps which should be taken.

Ten patients or parents reflected on their time in hospital. For some this was recent, for others longer ago. Whilst many were complimentary about the care they or their children received, and were appreciative of the opportunity to have innovative treatments not widely available, this report focuses on the perceived difficulties and shortcomings which have been experienced. In highlighting these, it would help give directions for services to be improved in the future. These have been grouped into themes.

Whilst information is given prior to obtaining comforters and carer’s consent, it is hard for parents to understand the level of risk they are exposed to when caring for a radioactive child. Further quantitative clarification of the levels of radiation exposure, and comparison of the risks associated with other forms of radiation exposure may be of value.

There can be little advance information about the duration of stay, and this makes planning for discharge more difficult, especially when long journeys need to be planned carefully. Better prediction of the likely fall in residual radioactivity levels to facilitate family arrangements would be beneficial.

There is concern about the risk of second cancers occurring in patients. Whilst this is a genuine risk, other factors including chemotherapy and genetic predisposition may also increase the risk. Mention of the risk of radiation carcinogenesis may cause unnecessary anxiety if the likelihood is overestimated, or if it is not communicated sensitively.

The reason for multiple post-treatment dosimetric scans is not always clearly understood by patients and parents, even when it has been explained carefully. The fact that these may show more areas of disease than prior diagnostic scans can be worrying. A clear explanation of the methodology and purpose of dosimetry is helpful. Reassurance that post-therapy uptake validation scans often show more ‘spots’ than were apparent on prior diagnostic imaging because of the greater activity injected may reduce unnecessary anxiety about disease progression.

Irregularity of radiopharmaceutical supply from the manufacturer adversely impacted four patients, whose treatment was delayed or rescheduled because of supply chain issues, which is a known European and international challenge [17]. Also, this was a major factor in the premature closure of the European multicentre clinical trial, VERITAS (NCT03165292). This trial was a groundbreaking evaluation of dosimetrically guided administration of ^131^I-meta iodobenzylguanidine (mIBG). Whilst it was recognised that this was beyond the immediate control of the hospital staff, hopes were expressed that a better dialogue with the companies involved might mitigate the problem in future.

Waiting lists for admission, sometimes many months long, meant that some patients did not receive treatment in a timely way, and there were concerns expressed that this might impact on the chances of a successful treatment. Clearly, greater capacity is needed.

Some staff groups do not work at weekends or public holidays, and it was suggested that if play specialists, therapeutic radiographers and radiation physicists who fully understood the service were available at this time, then difficulties experienced with ward medical and nursing staff might be lessened.

Whilst doctors and nurses were available on the ward for all necessary tasks, there was a perception that the radiation training of some was inadequate, meaning that they were possibly unduly concerned with looking after radioactive patients, resulting in families feeling abandoned within the protective suites – ‘out of sight and out of mind’ – with, for example, routine meal delivery being missed.

Facilities on the paediatric ward were felt to be not always age-appropriate, with teenagers in particular feeling out of place. Rooms were furnished with toys and decorations that were not always personalised and suitable for the age of the patient.

The importance of patient advocates in planning and supervision of clinical trials was stated to be important. It is helpful to get input at an early stage to ensure the design is as ‘patient friendly’ as possible, and to support successful research grant applications. Additionally, patient advocacy can help the results of successful trial lead to a more rapid change in standard of care treatments.

Eighteen responses were received, six from patients and parents. All said they felt comfortable sharing their experiences. Responses largely echoed what had been said during the meeting. Some selected ‘take home’ messages are shown in Table 3. Many respondents felt that it had been a valuable meeting. The majority are willing to continue to support paediatric MRT clinical service improvement and research. MRT for children is indicated for relatively small numbers of patients. Its delivery requires specially constructed suites with round-the-clock paediatric medical and nursing support, access to relevant imaging equipment for diagnosis, response evaluation, and dosimetric assessment (with appropriate anaesthetic support for young children), and experienced and dedicated staff. Most paediatric oncology principal treatment centres are unable to provide this, so the service, of necessity, is highly centralised.

**Table 3:** Responses to the questionnaire – selected ‘take home messages’

Patients and families are keen to know more about the practicalities of treatment.
There is interest in the treatments by many patients and families. There could be a greater understanding of the treatment. Why is treatment here different to the US? What is the difference between Lu-177 DOTATATE & I-131 mIBG (apart from availability!)
The inconsistency of the messages often being given to parents by the trial team, the radiology department and the ward staff. The inconsistency of the nursing on the ward. The lack of support for both the child and parent when in isolation. Both the patient and parent can feel ignored and left to get on with too much on their own under already trying circumstances.
Collaborative working is always beneficial to improve care for the future. This day has demonstrated the power that patient feedback, when shared in a constructive and positive way, can have on service improvement.
Connecting with other patients, parents and professionals in this area is important to share information. There has been a lot of work and improvements put into the treatment itself. Many people are willing to be involved in any further research etc. to help each other.

One potential weakness of this study was that all patients were treated in a single centre. Whilst some problems may be more widely experienced, others could be institution specific. Various deficiencies in the service provided in London were highlighted. Some related more to general paediatric care, and were not specific to MRT. Inadequacies in information provision and ward care issues should be straightforward to address. Work is underway to improve these aspects of service, and to provide more detailed and accurate information for families. Others, including security of radiopharmaceutical supply, waiting times and easier geographical access may be harder to resolve, although the professionals and charities involved are keen to raise awareness of these issues in appropriate local and national fora including national specialist societies and in meetings with industry and government representatives.

We plan to work more closely together in future, ensuring that professionals involved in this field, individual patient advocates, industry and charities such as Neuroblastoma UK [18], Solving Kids’ Cancer [19], Cancer Research UK [20] and the Children’s Cancer and Leukaemia Group [21], all of which both provide information for families and support research, collaborate to promote high standards of clinical service delivery and innovative research protocols in paediatric MRT.

PPIE can provide valuable insights into the experiences of those families requiring MRT. Inclusive collaboration between patients, parents and professionals can lead to productive relationships, improved patient care and research that addresses patient needs. We hope to have provided others with useful ideas to develop their own meaningful PPIE activities.

## Acknowledgements

We are grateful to Neuroblastoma UK, Solving Kids’ Cancer, Cancer Research UK City of London Centre Radiation Research Unit and Catapult Medicines Discovery for generous financial support which made this workshop possible, and to the Children’s Cancer and Leukaemia Group for administrative support. M.N.G. is supported by the National Institute for Health Research University College London Hospitals Biomedical Research Centre and by the Radiation Research Unit at the Cancer Research UK City of London Centre Award [C7893/A28990].

G.D.F. and M.N.G. contributed in the conceptualization. L.W. and C.P. contributed in the data curation. L.W., G.D.F. and M.N.G. contributed in the formal analysis. M.N.G. contributed in the funding acquisition. L.W. and M.N.G. contributed in the methodology. M.N.G. contributed in the project administration. Z.A. and L.K. contributed in the resources. L.W. contributed in the validation. M.N.G. contributed in the visualization. L.W. and M.N.G. contributed in the writing – original draft. All authors contributed in the investigation and writing – review & editing.

### Conflicts of interest

There are no conflicts of interest.
